# Editorial: Intraspecies variability in apicomplexan parasites: epidemiology, traits and virulence

**DOI:** 10.3389/fcimb.2024.1497043

**Published:** 2024-10-16

**Authors:** David Arranz-Solís, Debanjan Mukhopadhyay

**Affiliations:** ^1^ SALUVET Group, Faculty of Veterinary Sciences, Animal Health Department, Complutense University of Madrid, Madrid, Spain; ^2^ Institute of Health Sciences, Presidency University, Kolkata, India

**Keywords:** apicomplexa, *toxoplasma*, *neospora*, *eimeria*, *cystoisospora*, strain

Single cell eukaryotic obligate intracellular apicomplexan parasites are major cause of life-threatening infections in humans (*Plasmodium* spp., *Toxoplasma gondii*, *Cryptosporidium* spp.) and cause significant effect on cattle and poultry industries (*Neospora caninum* and *Eimeria* spp.) that leads to massive global economic loss every year. The outcome of infection and pathogenesis by apicomplexan parasites are largely dependent on the host and the parasite strain genotypes (Mukhopadhyay et al., 2020). In the current ‘omics’ era, with large datasets and new technologies frequently developed, the challenge is to combine these datasets to better understand the biological mechanisms by which parasites infect hosts and cause diseases, emphasizing the identification of key proteins that could be targeted for therapeutic or immunoprophylactic interventions. Due to the advent of the newer ‘game-changer’ methodologies, especially the CRISPR-Cas systems and the multi-omics driven approaches (whole genome sequencing, transcriptomics, proteomics, metabolomics, etc.), identification of newer strains and genotype-phenotype correlations became easier (Seeber and Steinfelder, 2016). In this sense, recent reports have highlighted that freshly obtained isolates have different phenotypic and genotypic traits compared to reference strains (Uzelac et al., 2024). In addition, new clinical signs and histopathological changes have also been described in different hosts or animal models (Sánchez-Sánchez et al., 2019). Finally, the distribution and epidemiology of each specific parasite is of relevance to understanding their importance in geographical areas that may be associated with outbreaks or cases with particularly unexpected outcomes.

In the present Research Topic, “*Intraspecies Variability in Apicomplexan Parasites: Epidemiology, Traits and Virulence*” published in Frontiers in Cellular and Infection Microbiology, we aimed to highlight the latest updates on the aforementioned Research Topic. Four articles have been published since June 2023 after a rigorous peer-review process by expert reviewers, focusing on four different apicomplexan parasites: *Cystoisospora suis*, *Eimeria necatrix*, *Toxoplasma gondii* and *Neospora caninum*.

The first work, entitled “*Unravelling the sexual developmental biology of Cystoisospora suis, a model for comparative coccidian parasite studies*” by Cruz-Bustos et al. provides novel proteomic insight into the life cycle of the enteropathogen of suckling piglets *Cystoisospora suis*. By performing LC/MS-MS analysis at five time points during the *in vitro* culture, the adaptation of sexual stages to a nutrient-poor and potentially stressful extracellular environment can be better understood. This model can serve as a valid tool to study the developmental biology of other related coccidian parasites such as *Emeria* spp. and *Toxoplasma gondii*.

In the second work entitled “*Localization in vivo and in vitro confirms EnApiAP2 protein encoded by ENH_00027130 as a nuclear protein in Eimeria necatrix*”, Cai et al. studied the Apicomplexan AP2 family of proteins (ApiAP2) in *Eimeria necatrix*, a parasite of importance for poultry worldwide. By focusing on EnApiAP2, encoded by ENH_00027130, authors describe the gene and protein features, as well as its localization in sporozoites and merozoites, confirming its role as a nucleoprotein. In addition, proteomic and transcriptomic analysis showed that this AP2 transcription factor is highly expressed in sporozoites. Overall, this work set the basis to better understand the role of AP2 proteins in *Eimeria* spp. development.

The third published work by Arranz-Solís et al., entitled “*A combination of GRA3, GRA6 and GRA7 peptides offer a useful tool for serotyping type II and III Toxoplasma gondii infections in sheep and pigs*” offers an easy and affordable non-invasive tool to perform initial epidemiology studies of the variability of *Toxoplasma gondii* genotypes present in sheep and pigs, two of the most relevant host species for *T. gondii*. The combination of polymorphic peptides from GRA3, GRA6 and GRA7 proteins were able to discriminate infections produced by type II or III strains in both pigs and sheep by ELISA. Further studies with more peptides and in other relevant host species will undoubtedly allow us to better study *T. gondii* epidemiology in livestock and design appropriate control strategies.

Finally, the work submitted by Román et al., entitled “*TaqMan-quantitative PCR assays applied in Neospora caninum knock-outs generated through CRISPR-Cas9 allow to determine the copy numbers of integrated dihydrofolate reductase thymidylate synthase drug selectable markers*”, further investigates potential usages of the cutting-edge CRISPR-Cas9 tools using *N. caninum* mutant strains as a model. Despite its undisputed importance in gene editing, CRISPR-Cas9 is not free of off-target effects, which can diminish its efficacy and cause unwanted effects in genetically modified strains. To determine the degree of off-target effects, authors used the common DHFR-TS resistance marker to study the number of integrated copies by means of a duplex qPCR in mutant strains. This tool provides a reliable and easy-to-use tool for assessing CRISPR-Cas9 mediated mutagenesis.

These four articles re-instated the importance of ‘multi-omics’ driven scientific facts in the identification and characterization of different virulence traits for better understanding of the apicomplexan biology that will help the ‘One-Health’ approach in a superior way. Before concluding this Research Topic, we editors would like to take the opportunity to thank all authors that submitted their work to this Research Topic, all the anonymous reviewers which provided a timely feedback and constructive input to ensure a high quality of the manuscripts, and the Frontiers editorial team for their guidance in setting up and managing this Research Topic. We hope that readers enjoy this Research Topic.
